# Evaluation of the accuracy of algorithms to identify soft tissue sarcoma (STS) in administrative claims

**DOI:** 10.1186/s13569-020-00130-y

**Published:** 2020-05-05

**Authors:** Nicole Princic, Donna McMorrow, Philip Chan, Lisa Hess

**Affiliations:** 1 IBM Watson Health, 61 Summer Avenue, Reading, Cambridge, MA 01867 USA; 2 Eli Lilly and Company, Indianapolis, IN USA

**Keywords:** Soft tissue sarcoma, Administrative claims, Methods, Coding

## Abstract

**Background:**

Lack of using a validated algorithm to select patients is a source of selection bias in oncology studies using administrative claims. The objective of this study to evaluate published algorithms to identify patients with soft tissue sarcoma (STS) in administrative claims and to evaluate new algorithms to improved performance.

**Methods:**

Two cancer populations including STS cases and non-STS controls were selected from the MarketScan Explorys Linked Claims-Electronic Medical Record (EMR) Database between January 1, 2000 and July 31, 2018. Eligible cases had a diagnosis on a clinical record for STS in the EMR while controls had no evidence of STS on any EMR records. Both cases and controls were enrolled in administrative claims during a period of observation and were aged ≥ 18 years. A split sample was used to test and validate algorithms using data from administrative claims. Values for sensitivity, specificity, and positive predictive value (PPV) were calculated for 14 algorithms. Prior literature validating algorithms in administrative claims across other cancer types report both sensitivity and specificity ranging from as low as 73% to as high as 95%. This was used as a benchmark for defining algorithm success.

**Results:**

There were 784 STS cases and 249,062 non-STS cancer controls eligible for analysis. Requiring at least two claims with an ICD-CM diagnosis code for STS achieved a sensitivity of 67% but had a specificity of 72%. Algorithms that required NCCN-recommended systemic treatment for STS improved the specificity to over 90% but dropped the sensitivity to below 20%. Other combinations of diagnostic tests, symptoms, and procedures did not improve performance.

**Conclusions:**

The algorithms tested in this study sample did not achieve sufficient performance and suggest the ability to accurately identify the STS population in administrative data is problematic. Difficulties are likely due to the origin of STS in a variety of locations, the non-specific symptoms of STS, and the common diagnostic tests recommended to diagnose the disease. Future research applying machine learning to examine timing and patterns of variables that comprise the diagnostic process may further investigate the ability to accurately identify STS cases in claims databases.

## Background

Soft tissue sarcoma (STS) is a rare malignancy of mesenchymal origin that develops in any of the soft tissues (e.g., fat, muscle, nerves, and blood vessels) of the body [1, 2]. There are over 50 subtypes of STS that can vary by molecular, histological, and clinical characteristics, making diagnosis and treatment challenging [2–4]. Approximately 43% of STS occurs in the limbs, 19% in the stomach and intestines, 15% in the retroperitoneum, 10% in the trunk, and 9% in the head or neck [5]. STS accounts for approximately 1% of all incident malignancies and an estimated 12,750 new soft tissue sarcomas were expected to be diagnosed in 2019 in the United States [6]. Surgical resection remains the main treatment of localized STS, however advanced STS requires a multimodal and multidisciplinary approach [5, 7].

Due to the large numbers of patients available in administrative claims databases, they are a valuable and commonly used real-world data (RWD) source for retrospective observational research. The validity of using administrative claims has been questioned, as important clinical prognostic factors (e.g., disease stage, histology) are absent and selection of patients is contingent on the accuracy of medical coding [8]. The International Classification of Diseases Clinical Modification (ICD-CM) system is used to code diagnoses in both inpatient and outpatient settings for billing and reimbursement purposes. Using ICD-CM diagnosis codes for health outcomes research is challenging as the codes often lack specificity needed to accurately identify patients [9, 10]. Prior work to validate the use of ICD-CM diagnosis codes in oncology research suggests there is variability in accuracy across different cancer types [11, 12]. One study developed an algorithm to identify multiple myeloma patients using administrative claims and found that requiring two diagnosis at least 30 days apart resulted in a sensitivity of 95% and a specificity of 73%. Requiring diagnostic tests, procedures, or chemotherapy treatment reduced the identification of false positives (specificity 94%) but reduced sensitivity to 83% [11]. A second study tested an administrative claims-based algorithm using a combination of diagnosis codes, procedures codes and treatments to identify patients with breast, colorectal, and lung cancer, the sensitivity of the algorithm was 77%, 72%, and 81% for each cancer type respectively [12].

A review of 64 oncology studies using administrative claims found that only 7% of studies used a validated algorithm to select patients, 36% used just one single claim with an ICD-CM diagnosis code [10], and only 5% discussed how the selection criteria could influence study findings. The diagnostic process for cancer is a complicated path with a combination of diagnostic tests and surgical procedures, therefore just one or two claims with the cancer diagnosis code may not be sufficient to minimize inclusion of false positives, however adding the requirement of specific pharmacologic treatment may be too restrictive (poor sensitivity). Lack of using a validated algorithm and the inaccuracies of using medical coding to select patients is a known source of selection bias in oncology studies using administrative claims databases [10, 13, 14].

Recently published literature suggests there is considerable variability in methodology used to identify patients with STS in administrative claims databases. Villalobos et al. required a National Comprehensive Cancer Network (NCCN) recommended pharmacologic therapy and at least two claims 30 days apart with an ICD-CM diagnosis code for STS [15]. Duh et al. used a similar approach, but identified patients using the specific ICD-CM diagnosis codes for STS or diagnosis codes for other cancer types in locations STS tumors are known to occur (e.g. retroperitoneal or peritoneal) in combination with NCCN recommended treatment [16]. Several other administrative claims-based studies identified patients using only ICD-CM diagnosis codes for STS with no specific treatment requirements [13, 17, 18]. Results from prior studies in STS using administrative claims have found treatment patterns to be inconsistent with recommendations and have identified a larger than expected proportion of patients with STS and no evidence of treatment [8]. These discrepancies raise concerns about the clinical significance of study findings and the accuracy of algorithms used to select patients. The objective of this study was to use linked claims and electronic medical records (EMR) to evaluate the sensitivity and specificity of published algorithms to identify patients with STS in administrative claims, and to evaluate the inclusion of additional factors could improve the ability to identify patients with STS in administrative databases.

## Methods

### Data sources

The IBM MarketScan Explorys Linked Claims-Electronic Medical Record (EMR) Dataset (CED) was used to conduct this study. This dataset links MarketScan claims with EMR data from two independent sources The MarketScan administrative claims databases and the Explorys EMR database. The MarketScan Commercial Claims and Encounters Database contains the inpatient, outpatient, and outpatient prescription drug experience of approximately 198.9 million employees and their dependents, covered under a variety of fee-for-service and managed care health plans, including exclusive provider organizations, PPOs, POS plans, indemnity plans, and health maintenance organizations (HMOs) between 1995 and 2018., including 25.9 million lives in 2018. The Medicare Supplemental and Coordination of Benefits Database additionally contains the healthcare experience (both medical and pharmacy) of approximately 14.3 million retirees with Medicare supplemental insurance paid for by employers between 1995 and 2018, including 1.1 million lives in 2018. The Explorys EMR database contains data for approximately 62 million patients integrated from 23 large health systems comprising approximately 360 hospitals and 330,000 providers. Data are collected from electronic health records, outgoing billing, and adjudicated claims from both commercial and public payers.

The CED linked dataset contains data for approximately 4.5 million patients derived from administrative claims and integrated health networks. The database provides a longitudinal view of patients’ medical histories, including clinical and economic data. Patients appearing in the claims-EMR linked files have a combination of the clinical detail from a variety of EMRs as well as the claims-level details of all provider visits, diagnoses, procedures, and medications. All database records are statistically de-identified and certified to be fully compliant with US patient confidentiality requirements set forth in the Health Insurance Portability and Accountability Act of 1996. Because this study used only de-identified patient records and did not involve the collection, use, or transmittal of individually identifiable data, Institutional Review Board approval to conduct this study was not necessary.

### Patient selection

Two cancer patient populations were identified from the CED between January 1, 2000 and July 31, 2018 (study period): STS cases (identified using systematized nomenclature of medicine [SNOMED] terms used to define STS on a clinical record in the EMR) and non-STS controls (patients with cancer but without evidence of STS in anyEMR records). SNOMED is a standardized, multilingual vocabulary of clinical terminology used by physicians and other health care providers for the electronic exchange of clinical health information. For inclusion in the case study sample, patients were required to have a SNOMED diagnosis for STS on a clinical record (not just a billing or historical record) in the EMR and be enrolled in the administrative claims database at the time of the initial STS diagnosis. All patients were required to have an ICD-CM diagnosis code for cancer (excluding osteosarcoma, Kaposi’s sarcoma, gastrointestinal stromal tumors or any hematologic malignancy) during the study observation period. For inclusion in the cancer control study sample, patients could not have any evidence of STS in the EMR during the entire study period. Eligible patients were required to have a period of overlap in which they were continuously enrolled in administrative claims and actively contributing data to the Explorys EMR. Cases and controls were excluded if they were less than 18 years of age at the start of the study period.

### Algorithm development and analysis

Following the selection of the STS cases and cancer controls, data were merged into one analytic file. A panel approach was adopted to test and modify published algorithms. For all cases and controls, panels were constructed around each eligible cancer diagnosis date (defined as the index date) to disenrollment in claims or the end of the study period for cases or during the longest period of overlap of enrollment in both claims and EMR during the study period for controls (observation period). The diagnostic period was defined as a minimum of the period 30 days prior to the index diagnosis through 180 days after index date. Once all eligible panels were identified, the file was randomly split (50/50) into a development sample and a validation sample. The validation sample was used for the highest performing algorithm(s). The split-sample approach allowed for statistical efficiency and unbiased estimates of the algorithm’s properties. Three algorithms (Algorithms #1, 2, and 4) were obtained from the literature [13, 15, 16] and represent the cohorts currently used to study patients with STS in claims data. Iterations of these algorithms were developed for a total of 14 tested algorithms as described in Table 1.

**Table 1:** Algorithms descriptions

Description
1) At least two medical claims with an ICD-CM diagnosis code for STS at least 30 days apart in any position [13]
2) At least two medical claims of any type at least 30 days apart with an ICD-CM diagnosis code for STS AND at least one claim for the prescription or administration of NCCN-recommended systemic therapy for STS treatment following the first STS diagnosis and within the diagnostic period [15]
3) At least one medical claim of any type with an ICD-CM diagnosis code for any non-STS solid tumor cancer AND No medical claims for any hematological cancers during the diagnostic period AND At least one claim for the prescription or administration of STS NCCN recommended regimen (single therapy or combination therapy identified by a 21 day window following the first treatment) on or following the diagnosis date and within the diagnostic period
4) At least one medical claim of any type with an ICD-CM diagnosis code for retroperitoneal or peritoneal cancer AND No medical claims with an ICD-CM diagnosis code for gastrointestinal stromal tumors, osteosarcoma, Kaposi’s at any time during the diagnostic period AND No medical claims for reproductive cancers at any time during the diagnostic period AND No medical claims for excretory cancer at any time during the diagnostic period AND No medical claims for cardio/pulmonary cancer at any time during the diagnostic period AND At least one claim for the prescription or administration of STS NCCN recommended regimen on or following the diagnosis date and within the diagnostic period [16]
5a) At least one medical claim of any type with an ICD-CM diagnosis code for STS and a second claim for STS any time after
5b) At least one medical claim of any type with an ICD-CM diagnosis code for STS and at least one claim for the prescription or administration of NCCN-recommended systemic therapy for STS treatment any time after
5c) At least one medical claim of any type with an ICD-CM diagnosis code for STS and at least one claim for an STS surgery prior to or following
5d) At least one medical claim of any type with an ICD-CM diagnosis code for STS and at least one claim for an STS symptom (pain in limb, localized superficial swelling mass lump, neoplasm or uncertain behavior in skin) prior
5e) At least one medical claim of any type with an ICD-CM diagnosis code for STS and (a second claim for STS following, or STS treatment following, or STS surgery prior to or following, or an STS symptom prior to)
6a) At least one medical claim of any type with an ICD-CM diagnosis code for STS, Ill-defined cancer, reproductive cancer, retroperitoneal or peritoneal cancer and a second claim for STS anytime following
6b) At least one medical claim of any type with an ICD-CM diagnosis code for STS, Ill-defined cancer, reproductive cancer, retroperitoneal or peritoneal cancer and at least one claim for the prescription or administration of NCCN-recommended systemic therapy for STS treatment following
6c) At least one medical claim of any type with an ICD-CM diagnosis code for STS, Ill-defined cancer, reproductive cancer, retroperitoneal or peritoneal cancer and at least one claim for an STS surgery prior to or following
6d) At least one medical claim of any type with an ICD-CM diagnosis code for STS, Ill-defined cancer, reproductive cancer, retroperitoneal or peritoneal cancer and at least one claim for an STS symptom prior
6e) At least one medical claim of any type with an ICD-CM diagnosis code for STS, Ill-defined cancer, reproductive cancer, retroperitoneal or peritoneal cancer and (a second claim for STS anytime following, or STS treatment following, or STS surgery prior to or following, or an STS symptom prior)

All variables used to develop algorithms were identified using the administrative claims databases and included imaging scans (i.e. computerized tomography, magnetic resonance imaging, radiograph, positron-emission tomography) surgical procedures (excision and resection), symptoms potentially related to STS (pain in limb, neoplasm of uncertain behavior in skin, localized superficial swelling mass or lump), sites of cancer diagnoses as defined by ICD coding (STS, gastrointestinal, head/neck area, nervous system, reproductive system, retroperitoneum/peritoneum, cardiopulmonary, excretory, endocrine, skin, ill-defined), and NCCN-recommended systemic therapies commonly used for STS (Table 2) [5].

**Table 2:** NCCN recommended single agents and combination regimens

Single agents
Doxorubicin
Ifosfamide
Epirubicin
Gemcitabine
Dacarbazine
Liposomal doxorubicin
Temozolomide
Vinorelbine
Eribulin
Trabectedin
Pazopanib
Regorafenib
Larotrectinib

Combination regimens
AD: doxorubicin, dacarbazine
AIM: doxorubicin, ifosfamide, mesna
MAID: mesna, doxorubicin, ifosfamide, dacarbazine
Ifosfamide, epirubicin, mesna
Gemcitabine and docetaxel
Gemcitabine and vinorelbine
Gemcitabine and dacarbazine
Doxorubicin and olaratumab (October 2016- July 2018 only)

Testing of algorithms started at the index cancer diagnosis date in each patient’s observation period and utilized the data within the diagnostic period. If the patient did not meet the algorithm criteria for STS at the first diagnosis, analysis moved forward to the subsequent cancer code in the patient record. A patient was identified as testing positive for STS per the algorithm at the earliest diagnosis that met all criteria. Algorithms #1, 2, and 5a-5e all required a claim with an STS specific ICD-CM diagnosis code followed by combinations of subsequent diagnoses, treatments, and procedures. Algorithms #3, 4, and 6a-6e did not require the specific STS ICD-CM diagnosis code but sought to identify STS patients using a combination of other factors (i.e. other cancer diagnosis, treatments, and exclusion conditions). Algorithms #2, 3, 4, 5b, 6a, and 6b required evidence of NCCN recommended systemic pharmacologic treatment for STS. For each algorithm, sensitivity, specificity, positive predictive value (PPV), and negative predictive value (NPV) were calculated. Prior literature validating algorithms in administrative claims across other cancer types report both sensitivity and specificity ranging from as low as 73% to as high as 95% [11, 12]. This was used as a benchmark for defining algorithm success.

## Results

### Study cohorts

**Fig. 1:**
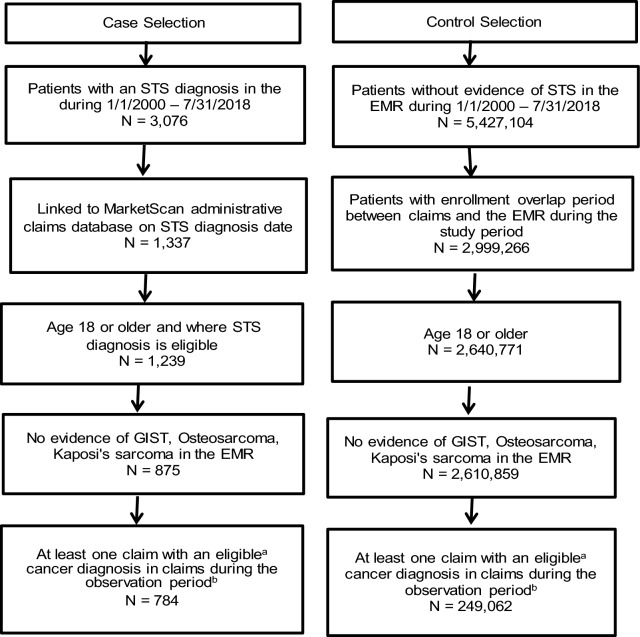
Case and control patient identification. **a** Eligible cancer diagnosis for control include all primary malignancies with the exception of STS, hematological malignancies, GIST, osteosarcoma, or Kaposi’s sarcoma. **b** For cases, the observation period is the number of days from the STS diagnosis in the EMR to disenrollment in claims or the end of the study period (7/31/2018). For controls, the observation period is the longest period of overlap of enrollment in both claims and EMR during the study period of 1/1/2000 through 7/31/2018

After patient selection, there were 784 STS cases and 249,062 non-STS cancer controls eligible for the analysis (Fig. 1). Among the eligible study population, STS cases had 21,746 panel index cancer diagnoses (10,906 in the development sample and 10,840 in the validation sample), and non-STS cancer controls had 3,601,216 (~ 1,800,000 each in the development sample and the validation sample) panel index cancer diagnoses available for testing algorithms. Tables 3 and 4 present the descriptive results of all demographic characteristics, symptoms, imaging scans, surgical procedures, treatments, and diagnoses around each index cancer diagnosis in the development sample for both STS cases and non-STS cancer controls. At index diagnosis, STS cases were younger (mean age 59.6 vs. 64.2) but had a similar gender distribution (54% female) compared with non-STS cancer controls (Table 3).. STS cases had a larger proportion with imaging scans and a higher number of scans prior to each index cancer diagnosis compared with non-STS controls. The majority of both STS cases and non-STS controls had a diagnosis code for a second non-STS cancer type (74.4% and 88.6%) following each index cancer diagnosis, and the mean (standard deviation, SD) number of other cancer diagnosis codes was 10.5 (15.2) and 14.5 (16.7) respectively (Table 4). Compared with controls, cases had a larger proportion of patients who received NCCN recommended pharmacologic treatment for STS prior to and following each index cancer diagnosis date, although the proportion with treatment was low in both groups.

**Table 3:** Cases and controls: development sample—demographics at index cancer diagnosis

	Controls N = 1,800,079	Cases N = 10,906
Age, mean (SD)	64.2 (12.7)	59.6 (14.8)
Age group, N (%)		
18–24	2958 (0.2%)	48 (0.4%)
25–34	21,453 (1.2%)	407 (3.7%)
35–44	89,631 (5.0%)	1280 (11.7%)
45–54	274,468 (15.3%)	2093 (19.2%)
55–64	569,910 (31.7%)	3345 (30.7%)
65–74	425,923 (23.7%)	1792 (16.4%)
75+	415,736 (23.1%)	1941 (17.8%)
Gender, N (%)		
Male	829,010 (46.1%)	4998 (45.8%)
Female	971,069 (54.0%)	5908 (54.2%)

**Table 4:** Cases and controls: development sample—diagnoses, treatments, procedures, symptoms at index cancer diagnosis

	Controls N = 1,800,079	Cases N = 10,906
Cancer diagnoses following index		
STS diagnosis, N (%)	11,054 (0.6%)	7590 (69.6%)
Number of STS diagnoses, mean (SD)	0.0 (0.9)	12.6 (20.0)
Non-STS cancer diagnosis, N (%)	1593,963 (88.6%)	8115 (74.4%)
Number of non-STS cancer diagnoses, mean (SD)	14.5 (16.7)	10.5 (15.2)
Treatments		
Any STS NCCN recommended treatment prior to index, N (%)	209,021 (11.6%)	2674 (24.5%)
Any STS NCCN recommended treatment following index, N (%)	171,301 (9.5%)	2548 (23.4%)
Days from index diagnosis date to treatment, mean (SD)	32.0 (42.0)	35.1 (43.6)
Regimen type, among those with treatment, N (%)		
NCCN recommended single agent	163,261 (95.3%)	1154 (45.3%)
NCCN combination regimen	2819 (1.7%)	1164 (45.7%)
Not a valid single agent or combination regimen	5221 (3.1%)	230 (9.0%)
Symptoms measured prior to index, N (%)		
Neoplasm of uncertain behavior in skin	117,979 (6.6%)	696 (6.4%)
Pain in limb	129,494 (7.2%)	1097 (10.1%)
Localized superficial swelling mass lump	15,017 (0.8%)	967 (8.9%)
Days from symptom to index, mean (SD)		
Neoplasm of uncertain behavior in skin	65.7 (49.7)	84.6 (50.2)
Pain in limb	76.0 (51.7)	74.6 (54.3)
Localized superficial swelling mass lump	79.9 (50.5)	89.4 (50.4)
Post diagnosis surgical procedures, N (%)		
Excision surgery	377,857 (21.0%)	2055 (18.8%)
Resection surgery	194,687 (10.8%)	1189 (10.9%)
Number of procedures, mean (SD)		
Excision surgery	1.4 (0.9)	1.4 (0.9)
Resection surgery	1.1 (0.4)	1.1 (0.5)
Pre-diagnosis surgical procedures, N (%)		
Excision surgery	485,480 (27.0%)	3552 (32.6%)
Resection surgery	285,294 (15.9%)	2341 (21.5%)
Number of procedures, mean (SD)		
Excision surgery	1.4 (0.8)	1.4 (0.8)
Resection surgery	1.1 (0.4)	1.1 (0.4)
Imaging scans (measured prior to or on index), N (%)		
Computerized tomography scan	1,051,001 (58.4%)	9401 (86.2%)
Magnetic resonance imaging	445,463 (24.8%)	4785 (43.9%)
Radiograph	1,135,315 (63.1%)	7797 (71.5%)
Positron-emission tomography scan	328,534 (18.3%)	3487 (32.0%)
Number of tests, mean (SD)		
Computerized tomography scan	3.7 (6.4)	4.5 (6.1)
Magnetic resonance imaging	1.4 (0.9)	1.5 (0.8)
Radiograph	2.3 (1.9)	2.5 (1.9)
Positron-emission tomography scan	1.2 (0.4)	1.4 (0.6)
Number of different scans, mean (SD)	4.1 (5.9)	6.8 (6.6)

### Algorithm performance

Table 5 presents a summary of performance of all tested algorithms. Algorithms (#2, 3,4, 5b, 6b) requiring STS NCCN pharmacologic treatment as a confirmatory criterion had a high specificity (91–99%) but very low sensitivity (< 20%). This low sensitivity was because only a quarter of cases received STS NCCN recommended systemic treatment. Algorithms #1 (which required two STS diagnoses at least 30 days apart), and 5a (which required two STS diagnoses on different days) had improved sensitivity (59% and 67%) but consequently the specificity dropped to 80% and 72% in both algorithms respectively. Algorithm #6e required a diagnosis of STS, Ill-defined cancer, reproductive cancer, retroperitoneal or peritoneal cancer diagnosis followed by a second STS diagnosis or STS NCC pharmacologic treatment, or an excision/resection surgical procedure or an STS related symptom achieved the highest sensitivity of 85% but specificity was below 40%. Algorithm #2 (which required two STS diagnoses at least 30 days apart and NCCN recommended systemic treatment) was the only algorithm that achieved a PPV over 50%. Modifications of published algorithms, through the inclusion of symptoms, procedures, and other cancer diagnosis codes did not improve algorithm performance (algorithms #5c, 5d, 5e and 6a, 6c, 6d). Given that none of the algorithms tested in the development sample achieved both sensitivity and specificity of 73% (i.e. the lower end of the acceptable range), validation using the second half of the study sample (“validation sample”) was not conducted.

**Table 5:** Algorithm test results

Short description	Algorithm	Sensitivity (%)	Specificity (%)	PPV (%)	NPV (%)
One STS diagnosis + a second STS diagnosis at least 30 days after the first	Algorithm 1	59.0	79.7	43.2	88.1
One STS diagnosis + a second STS diagnosis at least 30 days after the first + any NCCN recommended pharmacologic treatment for STS	Algorithm 2	16.4	97.0	58.8	81.6
One solid tumor cancer diagnosis + no evidence of hematologic cancer + an STS NCCN recommended single or combination regimen of pharmacologic treatment	Algorithm 3	2.2	99.5	1.5	99.7
One diagnosis of retroperitoneal or peritoneal cancer + no evidence of GIST, osteosarcoma, Kaposi’s + no reproductive cancer, no excretory cancer + no cardio/pulmonary cancer + an STS NCCN recommended single or combination regimen of pharmacologic treatment	Algorithm 4	15.4	97.0	24.0	95.0
One STS diagnosis + a second STS diagnosis any time after	Algorithm 5a	67.4	71.9	38.5	89.4
One STS diagnosis + any STS NCCN recommended pharmacologic treatment	Algorithm 5b	18.1	93.8	43.4	81.4
One STS diagnosis + surgery	Algorithm 5c	59.1	54.0	25.2	83.5
One STS diagnosis + symptom	Algorithm 5d	32.0	79.2	28.8	81.7
One STS diagnosis (+ second STS diagnosis or treatment or surgery or symptom)	Algorithm 5e	80.3	42.3	26.7	89.1
One STS, Ill-defined cancer, reproductive cancer, retroperitoneal or peritoneal cancer diagnosis + STS diagnosis following	Algorithm 6a	63.4	99.3	38.5	99.8
One STS, Ill-defined cancer, reproductive cancer, retroperitoneal or peritoneal cancer diagnosis + any STS NCCN recommended pharmacologic treatment	Algorithm 6b	19.0	91.5	1.5	99.4
One STS, Ill-defined cancer, reproductive cancer, retroperitoneal or peritoneal cancer diagnosis + surgery	Algorithm 6c	64.0	46.2	0.8	99.5
One STS, Ill-defined cancer, reproductive cancer, retroperitoneal or peritoneal cancer diagnosis + symptom	Algorithm 6d	36.0	76.8	1.0	99.4
One STS, Ill-defined cancer, reproductive cancer, retroperitoneal or peritoneal cancer diagnosis (+ second STS diagnosis or treatment or surgery or symptom)	Algorithm 6e	84.7	39.2	0.9	99.7

## Discussion

Since STS is a rare disease that can develop in any of the soft tissues in the body, it is challenging to diagnose [3, 19]. The study of patients with STS often relies on large real-world secondary data sources where sample size is sufficient [13, 15–18]. To our knowledge, this was the first study to develop and test algorithms to identify patients with STS in administrative claims. It was found that the algorithm requiring at least two claims with an ICD-CM diagnosis code for STS achieved a sensitivity of 67% but had a specificity of 72%. Therefore, using diagnosis codes alone is not sufficient, will lead to inclusion of false positives, and potentially erroneous results [8, 10]. Algorithms that required NCCN-recommended systemic treatment for STS improved the specificity to over 90% but dropped the sensitivity to below 20%. While this approach is more likely to find true cases with STS, it may not be a representative sample.

The inclusion of codes for other cancer diagnoses (i.e. other than STS) imaging scans, symptoms, and surgical procedures did not result in an algorithm with adequate sensitivity or specificity. Results from this study suggest that the ability to accurately identify the STS population in administrative data is problematic, likely due to the origin of STS in a variety of locations that may overlap with other diseases, the non-specific symptoms of STS, and the common diagnostic tests recommended to accurately diagnose the disease. These are health care resources that share coding structures identical to those of many other cancers. Prior literature validating algorithms in administrative claims across other cancer types report both sensitivity and specificity from 73 to 95% suggesting this range may be an acceptable standard [11, 12], but these levels were not achieved in this study. While the inclusion of NCCN-recommended therapies resulted in algorithms with specificity over 90%, the sensitivity was only 20% suggesting they still failed to meet sufficient performance standards.

The gold standard for evaluating algorithm performance in this analysis was a diagnosis of STS on the clinical record within the Explorys EMR, so limitations of EMR data should be considered when interpreting the findings. Patients are followed in the EMR systems only as long they come into the clinics and have billing records. Any care that patients may receive outside of the clinic cannot be captured. The impact of incomplete records and measurement error inherent in EMR databases should be considered as cases were not confirmed via pathology reports. This study was also limited to only those individuals that were linked between the IBM Explorys EMR data and the MarketScan Research Databases. Consequently, results of this analysis may not be generalizable to patients found in other administrative datasets.

## Conclusion

Accurate identification of a study population to reduce selection bias is an integral part of the study design in administrative claim-based studies across all tumor types and particularly problematic for STS [3, 13, 19]. A review published in 2013 found that only 12.5% of claims-based studies used a previously published algorithm and only 6.5% used a validated approach [10]. This analysis found that requiring two claims with a diagnosis code of STS may not be sufficient to limit false positives into the study population in administrative datasets, but further requiring disease-specific systemic treatment will exclude true sarcoma cases, resulting in a non-representative study sample. Given the limitations in identifying STS patients using administrative claims, results these studies using claims data alone should be interpreted with care. Future research using more automated statistical methodology such as machine learning methods such as classification and regression tree analysis to examine timing and patterns of diagnoses, procedures, testing, and symptoms that comprise the diagnostic process could potentially be used to better differentiate patients with STS from other cancer types more effectively in claims databases.

## Data Availability

All data generated or analyzed during this study are included in this published article. The data sources used in this analysis are proprietary and a license to use the data must be purchased.

## Abbreviations

EMR: Electronic medical records
GIST: Gastrointestinal stromal tumors
HIPAA: Health Insurance Portability and Accountability Act
ICD-CM: International Classification of Diseases, Clinical Modification
NCCN: National Comprehensive Cancer Network
NPV: Negative predictive value
PPV: Positive predictive value
RWD: Real World Data
SD: Standard deviation
STS: Soft tissue sarcoma
